# Round the Hospitals

**Published:** 1918-08-24

**Authors:** 


### ROUND THE HOSPITALS.

The Queen's visit to the hospital at Stratford which bears her name was an event of first importance to this institution. Her Majesty has taken a great interest in "Queen Mary's Hospital for the East End" ever since its change of name in 1916, for the fact that it does an enormous work for good among the "poorest of the poor" has secured her sympathy. Last year the casualties dealt with numbered 22,171, and the out-patients over 122,900. There are many wounded in the hospital and annexe to whom the Queen devoted her attention first of all, but under the guidance of the matron, Miss E. A. Sordy, A.R.R.C., she visited also the children's wards, the Duchess of Marlborough's women's surgical ward, the other civil wards, and the theatres and out-patients' department. A Royal Charter of Incorporation was bestowed on the hospital in December last. A new maternity block has been promised, and the great need now is for a really commodious nurses' home.

---

The Queen sets an admirable example to her subjects in the difficult art of visiting the sick in hospitals. Most matrons have wide experience of the ineptness of "visitors," who come at the wrong time, ignore the efforts made to welcome them,

**ROUND THE HOSPITALS—***(continued).*

hurry away, disappointing half the patients, and find nothing to say or do which can cheer the expectant sufferers. Her Majesty is always punctual; she has a trained eye for what is excellent; she never fails to admire the little tokens of welcome around her; she visits the entire establishment; she brings flowers so that all may have a pleasant memento of her visit. Best of all, she is at home with the patients, and knows how to make them feel that her interest is spontaneous and sincere. They enjoy the words she speaks to them, and soon begin to talk too. Her presence really cheers, and leaves everyone a little happier than before. Last week she conferred this great pleasure on the South London Hospital for Women, Clapham Common. Her visit to the Hospital for Sick Children, Great Ormond Street, was of a more intimate character, as its object was to see Princess Mary at work in her uniform in the Alexandra Ward. The Queen watched her daughter changing the dressings for one of the little ones, and could not resist the temptation of feeding one of the babes herself.

---

The Secretary of State for War has issued a list of over 800 matrons, sisters, staff nurses, and V.A.D. nurses who have been brought to his notice for valuable nursing services rendered in connection with the war. The hospitals from which these ladies have been singled out for mention are all in the Home area, and every branch of the Army Nursing Service is represented, while many civilian nurses now nursing the wounded are included. Included in the grand total are some forty members of the Canadian Army Nursing Service, two of the Australian A.N.S., and twelve of the New Zealand N.S., among whom we note the name of Miss Thurstan, the Matron-in-Chief. The list gives a wonderful impression of the devotion and skill placed at the service of the wounded in every part of the kingdom.

---

On August 15 a further list was issued of names, numbering in all nearly 800, which have been brought to the notice of the Secretary of State for War by the chairman of the Joint Committee of the British Red Cross Society and the Order of St. John of Jerusalem "for valuable services rendered in connection with the establishment, organisation, and maintenance of hospitals." The admirable services rendered in this connection by individuals in every part of the country undoubtedly deserve recognition, and this all the more because for the most part those thus singled out have not put themselves forward, but have laboured single-heartedly with others for the sake of the wounded.

---

The upward trend of salaries in hospitals is remarkably illustrated by the action of the General Committee of the North Staffordshire Infirmary. Prior to the war probationers at this hospital received £8, £10, £18, £25 in their first, second, third, and fourth years respectively, and sisters received £30, rising by annual increments of £2 to £40. At the end of the year 1916 this scale was increased. Staff nurses (fourth-year probationers) were raised to £35, and together with other probationers were accorded an annual war bonus of £2; the sisters had their salary raised by £10, commencing at £40 and rising by annual increments of £2 to £50. In addition, each sister after six months' service in this capacity was accorded a war bonus of £12 per annum. Many of the sisters receiving the war bonus of £1 per month are doing private work in the North Staffordshire Institute for Nurses, attached to the hospital, in which, after five years' service, the salary would amount to £50, plus £12 war bonus. Recently the scale has been again raised. Probationers now receive £12 in their first, £16 in their second, £20 in their third year; staff nurses receiving as before £35, with £2 war bonus. The revision has been done on generous lines, and the matron is to be congratulated on this accession of strength to the training-school.

---

The tireless labours of Mr. Basil E. Mayhew, I.C.A., in bringing order and control into the "Accounts of the Auxiliary Hospitals" should not be passed over without attention by the managers of civil hospitals. The table he gives showing the comparative cost of maintenance per day of patients both in different-sized hospitals and in 1916 and 1917 are fraught with instruction. The average cost of provisions worked out at 2s. 0½d. a day in 1916 and at 2s. 2d. in 1917, and it is noteworthy that "the smallness of the increase is accounted for chiefly by a much closer control over expenditure." As illustrating the importance of a penny in hospital finance it is shown that the increase of 1.34d. in the cost of maintaining a patient for one day in hospital during 1917 represents an additional expenditure of over £96,385 per annum. It is abundantly clear from the statistics collected that the process of recording the average daily or weekly consumption of various articles of food per head tends automatically and quite remarkably to reduce the cost. The practice of using "as little as possible" and trusting the cook "to allow no waste" is however still lingering in many hospitals.

---

It is greatly to be regretted that the nursing department should be practically ignored in the reports issued by many hospitals. When it is remembered how very large a share of the total expenditure in hospitals is necessarily spent on the nursing staff some information about the department appears due to the public. Hospitals as employers of labour have heavy responsibilities, and it is due to subscribers to learn how these are being met. Many ill-founded rumours, many idle slanders, would die a natural death in provincial towns if the rule were followed of giving the number of probationers admitted each year and the causes of failure to complete the course with the number of trained nurses certificated and the class of work undertaken by them. This is now done in establishments where

**ROUND THE HOSPITALS—(*continued*).**

modern requirements are studied, and the opportunity of mentioning any who have earned distinction is one which should be placed within the reach of every matron.

We note as a special feature in the Edinburgh Royal Infirmary Report a paragraph relating to domestic matters, usually entirely ignored by those who compile reports. The "Kitchen Mistress" (a charming title), Miss Steven, has retired after twenty-two years' service, having most ably conducted the management of the kitchen and trained an efficient assistant, Miss White, to take her post. When the hospital kitchen is brought back to its right place as the pivot on which the comfort of the whole establishment turns, it will no longer be an exception for the report to chronicle its successes. The Royal Infirmary does not disdain to tell us that "the reconstructed kitchen larders have proved a very great benefit," and the words breathe a spirit of good commissariat control.

Who says the little Auxiliary Red Cross Hospitals have been a failure? For structural reasons the one at Brynglas, Newport, South Wales, has been closed, and the seventeen wounded occupants have been transferred to fresh quarters. They write a touching "Round Robin" to the local paper recording their gratitude for their treatment, and we quote some sentences which might serve as the definition of "the ideal matron." "Although the matron has been strict with the patients she has been a mother to all of us. . . . No one can truthfully say she has made more of one than of the other, no matter what rank they held. She has shared our pleasures, joys, and sorrows. Those who have prayed for their lives on the battlefield know the way to pray for a blessing on others. Our one prayer now is that mother matron of Brynglas may long be spared to devote her untiring efforts to the boys in blue. Actions speak louder than words, yet we cannot speak too highly of her. We cannot praise her enough." Characteristically the name of the matron has been kept out of print. We seem to know that matron—the sort that always just manages to keep out of the limelight.

The memorial to Sister Medcalf which it is proposed to place in the chapel of Queen Charlotte's Hospital will enlist the support of a very large number of nurses and midwives who came under her influence. For twenty-four years she devoted herself with entire self-surrender to her duties in that institution. She trained a numerous section of the modern midwives who are now making history, and first as sister, and latterly since 1905 as assistant-matron, had a large share in setting the excellent tone for which Queen Charlotte's has long been famed. She was very devoted to the chapel and its services, and it has been fitly decided that the memorial shall take the shape of something which will enrich and beautify the spot with which it is linked by long association. Subscriptions, however small, will be welcomed by the matron, Miss Blomfield.

Nursing Sister A. Ross, who died at Buxton on July 12, was one of the pioneer band of nurses who came over from Winnipeg nearly four years ago with the first Canadian Contingent. For most of that time she served in France, at No. 1 General Hospital, but later she was transferred to this country, where some months ago her health broke down entirely. She had a high sense of patriotism, and was a devoted nurse. She was buried at Buxton Parish Church with full military honours, and over 150 nurses attended.

The French Flag Corps, one of the units of the British Committee of the French Red Cross, has been more than once singled out for distinction owing to the courage of its members. Recently the French Government has bestowed the Croix de Guerre on Miss Annie B. Mackinnon, one of the sisters attached to this branch of the Nursing Service, in recognition of the gallantry of the entire unit of her Corps close behind the French Front. Other members of the unit who have been mentioned in French dispatches are Miss Ellen Bennett, Miss Dora T. Simpson, Miss Annie B. Banks, Miss Lucy B. Giles, Miss Mary Richard, Miss Gladys Hawthorne. For the most part these sisters have been on active duty in French military hospitals since 1914. The Croix de Guerre has also been awarded to Miss Gladys Thompson, a V.A.D. nurse in a French military hospital, for bravery under fire and devotion to the wounded throughout the bombardment of her hospital.

The American Red Cross is beginning to experiment with the V.A.D. Major Perkins, Commissioner in Europe for the American Red Cross, has stated that the officials of this Society are much impressed by the splendid work done by our V.A.D. helpers, and are forming a body of their own. The first party of American V.A.D.s arrived in England on August 12, on their way to France. They wear a costume consisting of Norfolk jacket and skirt. The facings vary according to the class of work undertaken. It would seem that volunteer work is not always a success in America, if it be true that out of 900 leisured persons who offered their services, only one or two consented, when faced with a definite proposition, to undertake the required work.

The coal shortage this winter must be countered by vigorous methods in all institutions if the inmates are to be kept not only warm but clean. The problem of securing abundant supplies of hot water is at all times a serious one in a large household. It is frequently complicated by a faulty construction of hot-water cylinders and cisterns which allow of rapid cooling. To ensure thorough economy steps must be taken to heat the boiler

**ROUND THE HOSPITALS**—(*continued*).

with the least possible expenditure of fuel, a process involving often a complete overhauling of the system, and at the same time to ensure that the water remains hot. A current of cold air allowed to strike on the receptacle containing the hot-water supply will waste more in an hour than many baths. The remedy is to smear the cylinder or the cistern and the pipes exposed to draught with asbestos, and have them encased with match-boarding, a process neither difficult nor costly. Under this system water will remain hot day and night, and the cost of keeping a good supply is halved.

One of the ideals of Florence Nightingale was the establishment of a public service of women as obstetric surgeons; she deemed this branch of work to be particularly within the domain of women. It is interesting to find Mr. Hayes Fisher in his recent circular to County Councils and Sanitary Authorities advocating the replacement of the ordinary Inspector of Midwives under the Supervising Authority by qualified medical women. The functions of these qualified inspectors would be to visit each midwife in her area at least once a quarter and to instruct and advise her. Great dissatisfaction has been occasioned in the past by the appointment of inspectors ill prepared for their duties, and could the services of sufficient medical women be obtained for these posts and an adequate salary secured to them, there is no doubt midwifery would undergo a remarkable lift throughout the whole country. The gift of £10,000 bestowed by the American Red Cross on the Royal Free Hospital for the development of their new obstetric department comes exactly at the right moment, in view of this call for medical women.

It appears that there are 17,000 graduate nurses in America who have omitted to claim registration at the hand of the State. This is a curious commentary on what may be called the anti-social quality of the trained nurse. Because she does not see the good of it to herself, personally she fails to apprehend the benefit of State Registration to the profession at large. She remains outside the movement, and in so doing not only weakens her own position in ways which she is too ignorant to understand, but weakens the entire body to which she belongs. In planning the outline of a living registration system in this country, the great danger to guard against is wilful neglect on the part of nurses themselves to take advantage of it.

The Welfare Conference, convened by the Ministry of Munitions at Oxford on August 9, was largely occupied with the relationship of industrial fatigue and efficiency. They have discovered that long hours, especially in women, make for accidents. When the women worked seventy-five hours a week, or over twelve hours a day, accidents were three times more numerous than in a ten-hour day, and increased five-fold during the morning spell. Long hours, whether for men or women, do not yield the best output; this has long been known. The overworked nurses's actual output, the list of duties she performs in the wards, may not be dissimilar from that of her fresh and energetic sister working under better conditions. But, if measured critically by an expert, the value of the work would be seen to have undergone a subtle depreciation. The articles we have recently published on the hardening influence of the nurse's life have brought into strong relief the fact that over-fatigue stands first among those influences. It may be that close scrutiny of little accidents and lapses of memory in the nursing staff would reveal that long hours and inefficiency are nearly related in this direction also. The important thing is to determine what is the maximum per week which the nurse can habitually spend on duty without loss of efficiency or humanity.

An excellent suggestion that Parliament should grant the exclusive use of the name of "Nursing Sister" to every fully-trained, registered nurse for her protection, has been generally welcomed by the profession. It is suggested that sets of short popular leaflets, setting forth the moral and material attractions of the nurse's life, should be written. We have agreed to institute a Competition for the supply of these leaflets, and so give an opportunity to all interested to help in their preparation. Full particulars will be found in The Hospital of July 27, page 373. The conditions are as follows: 1. The leaflets must be short, from 1,000 to 2,000 words. 2. Leaflets should each deal with one branch, and one only, of nursing work, as, for instance, private nursing, fever nursing, village nursing, midwifery, and so on. Competitors may send in one or more leaflets. 3. Leaflets should be written for outsiders, and explain in easy language the conditions of training and future prospects of women who qualify for the branch of nursing selected, with the moral and material advantages of the life. 4. Intending competitors must send their names and addresses to the Editor of The Hospital, 28 Southampton Street, Strand, W.C. 2, by Sept. 1; the leaflets must reach him at the same address not later than Saturday, September 28, 1918. 5. Provided that thirty competitors enter and thirty leaflets at least are received, three prizes will be given of £3 3s., £2 2s., and £1 1s. We shall welcome 100 competitors at least. We hope our readers will freely compete, and so help the Editor, whilst they are thus helping themselves, to help the profession too.

☛ **It is our invariable practice to pay for all items of news which we may publish. The minimum payment is 5s. Paragraphs of about twenty lines in length—that is to say, of 150 words or less—are preferred. Contributions should be addressed to The Editor, The Hospital, 28 and 29 Southampton Street, Strand, London, W.C. 2, and, to be in time for the current week's issue, should reach him by the first post on Mondays.**

☛ For Mother and Child: Book Reviews, see p. 459; War Experiences and Adventures, see p. 460. The Women's Ambulance in a Shell Factory, p. 461.

